# Propensity Score Matching: Identifying Opportunities for Future Use in Nursing Studies

**DOI:** 10.3390/nursrep15050142

**Published:** 2025-04-27

**Authors:** Helena Blažun Vošner, Peter Kokol, Jernej Završnik

**Affiliations:** 1 Community Healthcare Center dr. Adolf Drolc Maribor, 2000 Maribor, Slovenia; helena.blazun@zd-mb.si (H.B.V.); jernej.zavrsnik@zd-mb.si (J.Z.); 2 Faculty of Electrical Engineering and Computer Sciences, University of Maribor, 2000 Maribor, Slovenia; 3 ECM Maribor, Alma Mater Europaea University, 2000 Maribor, Slovenia; 4 Medical Faculty, University of Maribor, 2000 Maribor, Slovenia

**Keywords:** nursing research, propensity score matching, synthetic thematic analysis, near-empty synthetic review

## Abstract

**Background:** The frequency of propensity score matching (PSM) use in research is exponentially increasing; however, its use in nursing has not yet been explored and is possibly underused. **Methods:** Synthetic knowledge synthesis has been used on two corpora of publications from the Web of Science bibliographic database for the following purposes: first, to identify the content of the current nursing PSM studies; second, to identify the content of nursing observational, retrospective, or other quasi-experimental studies; and finally, based on the above analyses, to explore new possibilities for further use of PSM in nursing. Findings: The use of PSM in nursing is very sparse, but the number and content of observational, retrospective, and similar nursing research is increasing and becoming more extensive. Ten prolific themes in observational nursing studies were identified. Based on these studies, several influential studies in which PSM has already been successfully used in comparable healthcare topics have been selected as opportunities for extended PSM use in nursing. **Conclusions:** As shown in the healthcare disciplines, the extended use of PSM in nursing research might make nursing research more consistent, relevant, internally and externally valid, and consequently more useful in clinical practice and research.

## 1. Introduction

With the rapid increase in the volume of real-world data and evidence [1], propensity score matching (PSM) has become widely used in quasi-experimental studies like retrospective analyses of healthcare datasets, registries, observational studies, and electronic medical record analyses based on individual patient covariates, equalizing potential confounding factors such as age, gender, or comorbidities when comparing different groups of patients. More precisely, the method attempts to adjust recognized unbalanced factors at baseline such that the data, once analyzed, will better approximate analyses performed on a prospective gold standard, namely randomized data (RCT) [2,3,4,5,6,7]. From the statistic perspective, in propensity score matching, pairs of patients are selected based on the difference between their propensity scores, and unpaired patients are dropped. Propensity scores are the distances between two patients computed from the covariates [8,9]. A propensity score is usually estimated by using the logistic regression model [10]. However, tree-based methods like bagging, random forests, and single classification trees [11] or recursive partitioning [12] have also been used. In addition to matching, propensity scores are also employed in a wider context [10,13], like stratification [14], inverse probability weighting [15], covariate adjustment [16], and conditional permutation inference [17].

PSM serves as a valuable retrospective statistical tool with several advantages. It has the potential to address ethical dilemmas encountered in RCTs, particularly those arising from the exclusion of specific patient populations, such as pregnant women and young children. This exclusion often results in a scarcity of robust clinical research validating treatment effectiveness for these groups, thus limiting the generalizability of findings to broader populations and enhancing external validity. When integrated with RCTs, PSM can yield superior research outcomes by allowing the two methodologies to complement each other effectively [18]. Nevertheless, PSM is not without its limitations. Two significant concerns arise: first, achieving a balanced sample does not necessarily ensure that the prognostic factors are equally distributed between groups; second, the process of matching may inadvertently lead to detrimental pruning if not executed properly, such as through the selection of inappropriate caliper widths or matching ratios [19]. Moreover, the application of PSM in small sample sizes can generate misleading results if utilized incorrectly [20]. Propensity scores are helpful in building matched pairs or strata that balance many observed covariates.

The use of propensity score matching in nursing research is much sparer than in medicine [21]. However, interesting results have been recently reported in 107 PSM nursing studies, for example, analyzing the impact of workforce violence against nursing staff [22], analyzing the impact of home and community care services pilot programs on healthy aging [23], analyzing the effects of special nursing units in nursing homes on healthcare utilization and cost [24], understanding the impact of chronic diseases on COVID-19 vaccine hesitancy [25], and analyzing if the treatment for insomnia symptoms is associated with reduced depression among older adults [26]. On the other hand, the number of observational, retrospective, and similar nursing studies where propensity score matching could be used is increasing, reaching more than 10,000 nursing studies in total in just the last five years.

The synthesis of PSM utilization in nursing research can be regarded as conducting either empty or near-empty reviews, first introduced by Lang et al. in 2007 [27] to prevent researchers from drawing unfounded implications for practice when confronted with a scarcity of eligible studies pertinent to a specific research inquiry. Moreover, Schlosser et al. [28] suggest that the existence of very few eligible studies may serve as empirical evidence highlighting the “need for future research”. The significance of near-empty reviews is further emphasized by the prevalence of such reviews within the Cochrane Database of Systematic Reviews [29]. Notably, several near-empty reviews have also been published within the nursing discipline [30,31].

In light of the aforementioned context and the limited number of PSM studies juxtaposed with a substantial body of observational nursing research, this study aims to conduct a synthetic knowledge synthesis [32] of relevant nursing and medical literature to explore potential avenues for the enhanced application of PSM in the nursing field.

## 2. Materials and Methods

To identify novel opportunities for using PSM within nursing research, we initially employed a synthetic knowledge synthesis methodology. This approach was utilized to conduct a comprehensive analysis of the thematic landscape of existing nursing studies employing PSM, alongside observational and retrospective nursing research. Through comparative analysis, we identified underutilized research areas where the application of PROs has been limited. Subsequently, these identified gaps were cross-referenced with analogous domains in medical research where PSM has demonstrated efficacy, with the aim of discerning transferable opportunities for advancing nursing scholarship. The execution of this process involved the following sequential phases:

1. Using synthetic knowledge synthesis, we identified study themes by analyzing publications describing PSM use in nursing published in the period 2020–2024 from the Web of Science bibliographic database, using the search string “*propensity score matching*” limited to the research area of nursing. The study period was limited to the recent five years to analyze state-of-the-art research and trends only. No additional inclusion/exclusion criteria were used.
2. Using synthetic knowledge synthesis, we identified the most popular themes for observational, retrospective, and other quasi-experimental studies in nursing using the same study period and bibliographic database as in Step 1. The search string was *observational or retrospective or “quasi?experimental”* limited to research area Nursing. No additional inclusion/exclusion criteria were used.
3. Comparing the themes identified in Step 1 and Step 2 and using themes emerging just in Step 2 as keywords, we searched for influential articles in the Web of Science and Scopus databases, where PMS has already been successfully used in medical applications. The cases presented in these articles were finally identified as new opportunities for PSM use in nursing.

Synthetic knowledge synthesis partially automates the knowledge synthesis process by triangulating bibliometric mapping and content analysis. It enables one to synthesize large corpora of publications by visualizing the relationships and associations between units of analysis, thus simplifying the naming of categories and themes, and reducing the time and resources needed [32]. This enhanced efficiency enables synthetic knowledge synthesis to analyze large-scale bibliographic datasets (whole corpora of publications), circumventing the sampling challenges inherent in traditional knowledge synthesis approaches, like systematic or scoping reviews, where synthesis is applied only on a small sample of publications. Such sampling limitations often restrict synthesis outcomes to narrow topics while overlooking broader patterns. Consequently, the identification of sample publications used in traditional synthesis approaches might be biased, leading to non-reproducible syntheses [33]. Moreover, synthetic knowledge synthesis leverages triangulation to generate a holistic understanding of phenomena, enhancing the validity and comprehensiveness of the synthesis. From the processing point of view, synthetic knowledge synthesis is performed using the algorithm outlined below:

1. Develop a comprehensive search strategy to compile a relevant corpus of publications that addresses the research objectives through a knowledge synthesis process.
2. Select Author Keywords as units of information for content analysis, as they precisely reflect the intended focus of the research that authors aim to share with the academic community, while maintaining a balance between structured terminology and author-driven expression.
3. Perform a bibliometric mapping of author’s keywords into a clustered bibliometric map using VOSViewer [34].
4. Analyze the links and proximity between author keywords in individual clusters to form categories.
5. Condense categories into themes.

In this study, VOSViewer was employed to perform bibliometric mapping, VOSViewer uses text mining, co-word analysis, and clustering algorithms to identify connections between analytical units, in our case, author keywords. The resulting visualizations display author keyword relationships as links, with clusters of related keywords, each represented by a unique color, indicating categories and themes.

## 3. Results and Discussion

### 3.1. Synthetic Knowledge Synthesis of Nursing PSM Studies

**Figure 1:**
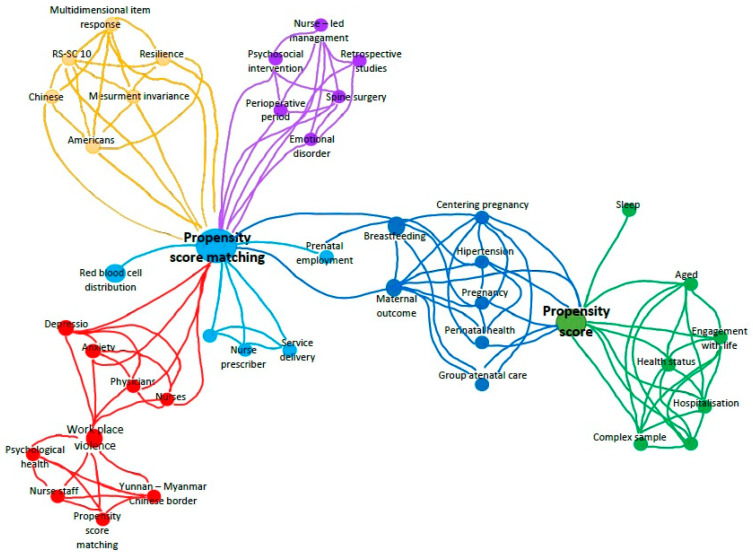
The author’s keywords landscape of PSM use in nursing research for the period 2020–2024.

For the period 2020–2024, only 76 papers describing the use of PSM in nursing were found. Comparing that number to almost 10,000 PSM studies in healthcare, we can safely conclude that PSM use in nursing is extremely sparse. The bibliometric mapping of those 76 studies performed with VOSViewer resulted in the author’s keyword landscape presented in Figure 1. The landscape consists of six clusters, which were analyzed using content analysis. The links between each cluster and the content analysis of the landscape revealed the following themes:

- *Red cluster: Psychological health (anxiety and depression) of nursing staff after workplace violence*. PSM and regression analysis were used to compare depression and anxiety symptoms in physicians and nurses who had or had not experienced workplace violence [35] or whether workplace violence affects psychological health in general [22].
- *Light blue cluster: Nurse-led management* [36].
- Yellow: Resilience and regret in cancer patients: PSM was used to assess measurement invariance in using resilience instruments in cancer patients [37] and regret in parents of children with cancer [38].
- *Violet cluster: Nurse-led management*. PMS was used to compare groups of patients who received nurse-led multidisciplinary psychological management and who did not [36] to compare anesthesia-related outcomes between patients monitored by newly recruited nurse anesthetists and those monitored by newly recruited anesthesiologists [39].
- *Dark blue cluster: Group antenatal care*. PSM was used to assess the effects of pregnant women in participating in Centering Pregnancy on maternal, birth, and neonatal outcomes [40] and compare antenatal complications in obese and non-obese women [41].
- *Green cluster: Successful aging*. PSM was used to assess the effect of hospitalization on successful aging [42].

### 3.2. Syntetic Knowledge Synthesis of Nursing Observational, Retrospective and Other Quasi Experimental Studies

**Figure 2:**
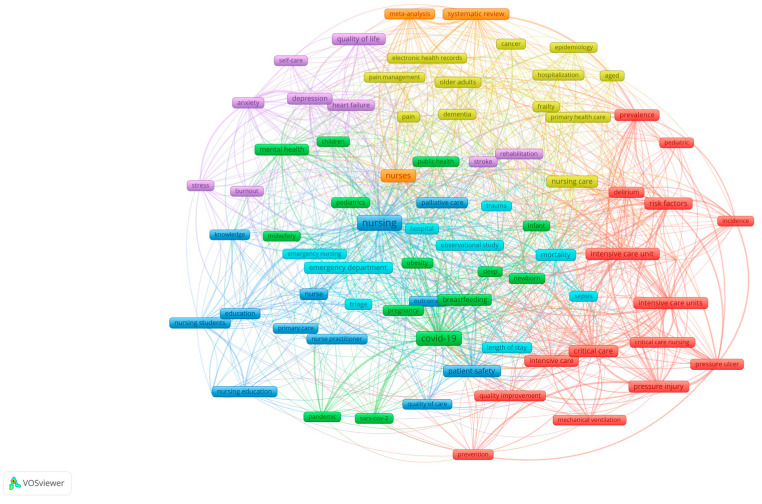
The author’s keyword map of observational, retrospective, and other quasi-experimental nursing studies in the period 2020–2024.

For the period 2020–2024, 9463 publications reporting results from observational, retrospective, and other quasi-experimental nursing studies were harvested. The author’s keyword landscape of these studies is shown in Figure 2. The content analysis performed on the landscape shown in Figure 2 resulted in the following themes:

- Nursing education;
- Emergency and critical care nursing;
- Primary care nursing;
- Patient safety and quality of care;
- Pandemics;
- Midwifery;
- CVD rehabilitation;
- Quality of life and self-care/management for all ages;
- Pain management;
- Epidemiology from the nursing perspective.

### 3.3. Identifying a Sample of New Opportunities

While the analyses presented herein demonstrate a limited application of propensity score matching (PSM) in contemporary nursing research, the thematic breadth of observational, retrospective, and quasi-experimental nursing studies is considerable. A critical examination of the thematic distribution reveals a distinct divergence between studies employing PSM and those utilizing observational, retrospective, or quasi-experimental methodologies. This divergence highlights a significant opportunity to extend PSM’s application within nursing research. To capitalize on these opportunities, an extensive search in WoS and Scopus bibliographic databases was undertaken to identify exemplary studies employing PSM in analogous health-related fields. The aim of this review was to discern suitable thematic areas for potential PSM integration in nursing, thereby enhancing the rigor and validity of future research. The results of this review indicating themes and suitable references are presented in Table 1.

### 3.4. Study Limitations

This research study did have some limitations. First, the use of Web of Science Core Collection as the only bibliographic data source could have omitted relevant literature from the analysis. The interpretation of the author’s keyword landscape was qualitative and consequently subjective. The search was limited to nursing journals only; thus, some publications might be overlooked. However, a comparable search “Propensity score matching” AND Nursing resulted in 113 publications only which still reflects the underuse of PSM in nursing discipline. Readers should also note that the concept of a propensity score represents a broader methodological framework beyond its application in matching techniques. In the analyzed articles that reference “propensity score”, alternative procedures might also be used. Finally, this paper is a Communication with the main aim of informing the nursing community about PSM and the possible opportunities where it can be successfully used.

## 4. Conclusions

Unlike general healthcare research, PSM is rarely used in nursing. The most likely reason is the lack of awareness among nursing researchers about its benefits and potential applications. Additionally, the majority of nursing studies are qualitative, and there might be a lack of advanced statistical training in nursing staff and a lack of comprehensive nursing-related datasets. However, successful PSM use in healthcare shows that there are many comparable nursing research scenarios where PSM might be successfully employed. In this way, observations, conclusions, and new knowledge gained from observational, retrospective, and other nursing quasi-experimental studies might become more consistent, relevant, internally and externally valid, and consequently, more useful and evidence-based in clinical practice and research. In addition to the above opportunities, future PSM use in nursing might be used in investigating complex nursing interventions and their outcomes, addressing health disparities by controlling confounding variables that influence patient outcomes across different, or even combining PSM with other methods like casual mediation analysis or randomized nursing clinical trials.

## Figures and Tables

**Table 1:** Opportunities for PSM use in nursing.

Themes of Nursing Observational Studies	Opportunities Translated from General Healthcare Research Where PSM was Used
Nursing education	Comparing distance/blended and face-to-face learning [43,44], does voluntary clinical practice improve study outcomes [45]?
Emergency and critical care nursing	Impact of personal protective equipment [46].
Primary care nursing	Effectiveness of self-management in the elderly [47], effectiveness of diets in chronic diseases [48].
Patient safety and quality of care	Patient safety and efficiency of the health of robots [49].
Midwifery	The effects of midwifery continuity care on delivery [49], association of quality of care with healthcare costs [50].
CVD rehabilitation	Effectiveness of early rehabilitation in intensive units [51].
Quality of life and self-care/management	Association between self-medication for mild symptoms and quality of life among older adults [52], long-term effects of severe COVID-19 [53].
Pain management	Effect of perioperative pain neuroscience education [54], impact of biological sex patients with chronic pain [55].
Epidemiology	Emulating randomized clinical trials with nonrandomized real-world evidence studies [56], association between initial treatment strategy and long-term survival [57].

## Data Availability

No new data were created or analyzed in this study. Data sharing is not applicable to this article.

## Public Involvement Statement

No public involvement in any aspect of this research.
